# Body Image Scale: Evaluation of the Psychometric Properties in Three Indian Head and Neck Cancer Language Groups

**DOI:** 10.3389/fpsyg.2022.779850

**Published:** 2022-05-12

**Authors:** Chindhu Shunmugasundaram, Haryana M. Dhillon, Phyllis N. Butow, Puma Sundaresan, Mahati Chittem, Niveditha Akula, Surendran Veeraiah, Nagraj Huilgol, Claudia Rutherford

**Affiliations:** ^1^Centre for Medical Psychology and Evidence-Based Decision-Making, School of Psychology, Faculty of Science, The University of Sydney, Sydney, NSW, Australia; ^2^Psycho-Oncology Cooperative Research Group, School of Psychology, Faculty of Science, The University of Sydney, Sydney, NSW, Australia; ^3^Radiation Oncology Network, Western Sydney Local Health District, Sydney, NSW, Australia; ^4^Sydney Medical School, Faculty of Medicine, The University of Sydney, Sydney, NSW, Australia; ^5^Department of Liberal Arts, Indian Institute of Technology Hyderabad, Sangareddy, India; ^6^Department of Psycho-Oncology, Cancer Institute (WIA), Chennai, India; ^7^Division of Radiation Oncology, Nanavati Super Speciality Hospital, Mumbai, India; ^8^Quality of Life Office, School of Psychology, Faculty of Science, The University of Sydney, Sydney, NSW, Australia; ^9^Susan Wakil School of Nursing and Midwifery, Cancer Nursing Research Unit, The University of Sydney, Sydney, NSW, Australia

**Keywords:** validation, cross-cultural adaptation, body image, head and neck cancer, Indian languages

## Abstract

**Background:**

Body image is a subjective concept encompassing a person’s views and emotions about their body. Head and neck cancer (HNC) diagnosis and treatment affects several psychosocial concepts including body image. Large numbers of HNC patients are diagnosed each year in India but there are no suitable measures in regional languages to assess their body image. This study assessed the psychometric properties of the Body Image Scale (BIS), a measure suitable for clinical and research use in HNC populations, translated into Tamil, Telugu and Hindi and compared body image distress between language groups.

**Methods:**

Translated versions of BIS were completed by HNC patients recruited from three cancer centers across India one time only. Psychometric evaluation was conducted including factor analysis using principal component analysis and internal consistency reliability using Cronbach’s alpha. Patients completed the EORTC Quality of Life Questionnaire (QLQ) C-30 and EORTC QLQ HN-35 measures to enable exploration of convergent and discriminant validity. ANOVA was used to calculate difference in mean values for body image.

**Results:**

Our sample included 621 HNC patients (Tamil = 205, Telugu = 216, Hindi = 200). Factor analysis revealed a one-factor solution and Cronbach’s alpha coefficients ranged between 0.891 and 0.969 indicating good reliability. Hypothesized correlations between similar and different constructs were as expected, supporting construct validity. On the BIS, we found a statistically significant difference (*F* = 11.0954, *P* < 0.05) between means of Tamil, Telugu, and Hindi groups, with higher body image scores in Telugu (*M* = 12.86; *SD* = 7.65) and Hindi groups (*M* = 12.52; *SD* = 7.36) indicating more symptoms/body image distress, when compared to Tamil population (*M* = 9.28; *SD* = 10.04).

**Conclusion:**

The reliability and validity of the three translated Indian versions of the BIS were maintained, providing a method for assessing body image of HNC population worldwide speaking Tamil, Telugu, and Hindi across the illness trajectory.

## Introduction

Head and neck cancers (HNC) in India are a major health problem, constituting about one-third of all cancers (Shah et al., 2016). According to Global Cancer Incidence, Mortality, and Prevalence (GLOBOCAN), over 100,000 cases of oral malignancies are registered in India each year (Johnson and Amarasinghe, 2016; Bray et al., 2018). Main causes of HNC in India are chewing tobacco, smoking bidis and cigarettes, and alcohol consumption. Regardless of clinical advances in early diagnosis and treatment, HNCs result in significant functional, physical, and psychosocial effects and affect mortality rates (Gupta et al., 2016). Treatment toxicity is common and can lead to scarring, appearance changes or disfigurement, difficulties in talking, eating and swallowing, sticky saliva, weight loss, and dental problems (Semple et al., 2008). People treated for HNC most commonly report body image concerns from disfigurement and scarring. Studies have shown poorer body image in HNC patients post-surgery compared to before surgery (Hung et al., 2017) and that reduced body image is associated with a decline in general health-related quality of life (HRQoL) (Nayak et al., 2016; Manier et al., 2018).

Physical appearance and attractiveness are highly valued by individuals (Sharma et al., 2018) but influenced by various psychosocial factors such as personality, interpersonal factors (family and peers), and social factors (values and norms) (Sharma et al., 2018). Generally, when a person is diagnosed with a life-threatening disease such as cancer, they may initially perceive their physical appearance and body image as less important than concerns about survival (Page and Adler, 2008). Some individuals may continue to perceive disfigurement positively, reporting gratitude for being alive and surviving cancer, and self-confidence in dealing with body changes (Manier et al., 2018). While others may come to perceive disfigurement as an embarrassment resulting in social withdrawal, isolation, feelings of inadequacy, anxiety, and low self-esteem, all consequently contributing to poor HRQoL (Sundaram et al., 2019). Addressing body image issues may prevent these negative outcomes (Sharma et al., 2018). Therefore, in a population susceptible to disfigurement or appearance related changes, it is crucial to assess patient-reported outcomes (PROs) such as body image throughout the HNC disease and treatment journey to identify concerns and provide appropriate supportive care (Hopwood et al., 2001).

Many patient-reported outcome measures (PROMs) have been developed in English and are not suitable for use in countries where people do not speak English, or in immigrant populations or minority groups who also may not speak English (Guillemin et al., 1993; Saxena et al., 1998). Furthermore, with increased global immigration, an individual country can have inhabitants whose linguistic and cultural origins vary widely (Castles, 2000). With increasing transnational research involving multiple countries, including diverse patient groups in research is crucial as without adequate representation, the generalizability of study results to all segments of the population is questionable. There is potential for systematic bias in studies if whole sections of a population are excluded because they do not speak English or measures are not appropriate for them (Anderson et al., 1993; Beaton et al., 2000). Therefore, culturally relevant, valid, and reliable PRO measures are essential.

Translating existing valid and reliable patient-reported outcome measures (PROMs), rather than developing new ones, reduces effort, saves time, and speeds up the acquisition of knowledge related to cultural differences (Guillemin et al., 1993). To use a PROM across cultures, the items in the measure should be translated, their content validity and cultural appropriateness assessed (Sperber, 2004), and psychometric properties such as reliability, validity, and responsiveness tested. Such cross-cultural adaptation helps achieve equivalence between the original and translated versions of a measure (Guillemin et al., 1993).

India being a large, culturally diverse country, has an increasing number of clinical trials being conducted because of its potential to recruit large patient samples (Saxena et al., 1998; Gupta and Padhy, 2011). However, primary reasons reported for excluding Indian populations from participation in research are illiteracy and language barriers, with the main concern being lack of psychometrically robust PROs. Some PRO measures specific to HNC have been translated into few Indian languages and are cross culturally validated (Pandey et al., 2004; Thomas et al., 2004; Chaukar et al., 2005, 2009). For example, HNC-specific measures of quality of life developed by the European Organization for Research and treatment of Cancer Quality (e.g., EORTC QLQ-HN35) and the Functional Assessment of Cancer Therapy (FACT – Head and Neck) have been translated into several regional Indian languages (Chaukar et al., 2005). However, those assessing body image are not currently available in common Indian languages (Sundaram et al., 2019). Our earlier work found the Body Image Scale (BIS) to be the most appropriate measure for assessing body image in HNC populations following appraisal of its content and psychometric properties (Sundaram et al., 2019).

Our team recently translated the BIS into the Indian languages Tamil, Telugu, and Hindi, and demonstrated their cultural appropriateness and conceptual equivalence with the English original (Shunmugasundaram et al., 2021). The purpose of this study was to analyze the psychometric properties of the translated Indian versions of the BIS in HNC patients who speak Tamil, Telugu, and Hindi.

## Materials and Methods

This study is a part of a larger study evaluating the psychometric properties of several translated PROMs assessing body image, unmet needs, anxiety, and depression in Indian HNC patients (Shunmugasundaram et al., 2020, 2021).

### Sample Eligibility

Patients were eligible if aged 18 years or above, any gender, diagnosed with any type of HNC except thyroid cancer (irrespective of cancer stage or treatment phase), treated by surgery, radiotherapy, chemotherapy, or a combination of these, could read and write in one or more of the target languages: Tamil, Telugu, and Hindi, and gave informed consent.

### Study Sites

This study was carried out in three regional cancer centers in Tamil Nadu (Cancer Institute, WIA in Chennai for Tamil speaking patients), Andhra Pradesh and Telengana (MNJ Institute of Oncology and Regional Cancer Centre, Hyderabad for Telugu speaking patients) and the Nanavati Super Specialty Hospital, Mumbai (for Hindi speaking patients) between August 2019 and February 2020.

### Recruitment and Consent Process

Eligible patients in participating cancer hospitals were informed about the study by a member of their clinical team (oncologist, psycho-oncologist, nurse, or ward assistant). Patients who expressed interest in the study were then approached by trained researchers who explained the study in detail and obtained participants’ written consent. A minimum of 600 patients (200 from each language group) meeting the eligibility criteria were approached by researchers. We sampled purposively but broadly to ensure representation of a wide range of HNC types, treatment types, disease stages, and impacts. Recruitment was periodically monitored for these variables.

### Sample Size

Sample size was estimated based on recommendations in the literature (Tabachnick and Fidell, 1996; Pallant, 2013), suggesting 5–10 participants per item in the measure being evaluated. Hence, a sample size of 50–100 participants would be required. However, larger samples are required for factor analyses (Comrey and Lee, 1992; Thompson, 2004). Therefore, we considered 200 participants per language group fulfilling the criteria and would provide an adequate sample to evaluate standard psychometric properties.

### Ethics

Ethics approvals were obtained from The University of Sydney Human Research Ethics Committee (Sydney, Australia), Scientific Advisory Committee and Ethical Committee of Cancer Institute, WIA (Chennai, India), Ethical Committees of MNJ Institute of Oncology and Regional Cancer Centre, Hyderabad and Nanavati Super Specialty Hospital, Mumbai, India.

### Data Collection

Participants self-completed hard-copy questionnaire booklets that included demographic information as well as three PROMs. Participants were asked to return their booklets to the researcher on completion of the PROMs. Data were entered into a REDCap (Harris et al., 2009) database by one researcher and manually checked for errors by a second researcher to ensure accuracy.

### Patient-Reported Outcome Measures

#### Body Image Scale

The 10-item BIS was developed to assess changes in body image in patients diagnosed with cancer, irrespective of their diagnosis, assessed with a four-point scale from “not at all” to “very much.” High scores indicate higher body image. The BIS has been shown to be reliable (Cronbach’s alpha 0.93), and valid (Hopwood et al., 2001). The BIS was translated from English to three Indian languages (Tamil, Telugu, and Hindi) following internationally accepted methods, ensuring conceptual and linguistic equivalence was maintained between versions (Shunmugasundaram et al., 2021).

#### Health-Related Quality of Life

The European Organization for Research and Treatment of Cancer (EORTC) Quality of Life Questionnaire (QLQ-C30) is a 30-item measure of cancer-specific symptoms and quality of life relevant to a broad range of cancer populations (Aaronson et al., 1993). It includes five functional domains (physical, role, cognitive, emotional, and social), eight symptom domains (fatigue, pain, appetite loss, constipation, sleep, dyspnea, diarrhea, nausea, and vomiting) and a global health/quality of life and financial impact domain. Items 1 to 28 are assessed with a four-point scale from “not at all” to “very much” and items 29 and 30 are assessed with a 7-point Likert scale from “very poor” to “excellent”. Raw scores are transformed to a 0–100 scale and higher scores represent better functioning and greater symptom burden. The EORTC QLQ-C30 has been shown to be reliable (Cronbach’s alpha ≥0.70) and valid (Aaronson et al., 1993), and is available in several languages including Tamil, Telugu, and Hindi, which were used in this study.

The EORTC QLQ Head and Neck 35 (EORTC QLQ-HN35) is a 35-item measure of HNC-specific symptoms (Bjordal et al., 1999). It includes 18 symptom domains (pain, swallowing, teeth, opening mouth, dry mouth, sticky saliva, senses, coughing, felt ill, speech, social eating, social contact, sexuality, pain killers, nutritional supplements, feeding tube, weight loss, and weight gain). Items 31–60 are assessed on a 4-point scale from “not at all” to “very much” and items 61–65 are assessed on a yes or no scale. Raw scores are transformed to a 0–100 scale and higher scores represent greater symptom burden or problems (Bjordal et al., 1999). The EORTC QLQ-HN35 has been shown to be reliable (Cronbach’s alpha ≥0.70) and valid (Bjordal et al., 1999; Bjordal et al., 2000). EORTC QLQ-HN35 is also available in several languages including Tamil, Telugu, and Hindi.

All PROMs in our study have previously been used in other studies of HNC patients (Chaukar et al., 2005).

### Data Analysis

The psychometric analyses of the BIS were undertaken using Statistical Package for the Social Sciences Version 25.0 (IBM Corporation Released 2017. IBM SPSS Statistics for Windows, Version 25.0. Armonk, NY, United States: IBM Corporation). Descriptive statistics (number and percentage of total sample) reported sample characteristics across three languages. Data quality and completeness were assessed based on percentage of computable scale scores (>50% completed items).

Exploratory factor analysis using principal component analysis with varimax rotation was performed to identify the underlying factor structure of the BIS. Factors with eigen value <1 and items with loading of minimum 0.3 were retained. Missing values were managed using pairwise deletion. The suitability of the data for factor analysis was assessed using the Kaiser–Myer–Olkin (KMO) measure of sampling adequacy and the Bartlett test of sphericity. Criteria for suitability are KMO 0.6 and a *P*-value for Bartlett’s χ2 of less than 0.01 (Moreira et al., 2010; Pallant, 2013). Confirmatory factor analysis (CFA) was carried out using Lavaan and Structural equation modeling (SEM) Package in R Commander. The goodness-of-fit indices were examined without any limitations or adding new connections (Hu and Bentler, 1999).

Internal consistency reliability was assessed using the Cronbach’s alpha coefficient for each of the language groups. A Cronbach’s alpha coefficient of 0.7 or greater was considered acceptable (Gorecki, 2011).

Scale-to-sample targeting was determined by investigating whether scale scores spanned the entire scale range; floor (proportion of the sample at the maximum scale range) and ceiling (proportion of the sample at the minimum scale range) effects were low (<15%). Scaling assumptions were assessed based on item-total correlations (ITC) where ITC ≥0.3.

Construct validity of the BIS was explored through correlations between body image (assessed with BIS) and other constructs (similar and dissimilar) assessed by the EORTC QLQ-C30 and EORTC QLQ-HN35. Pearson’s correlation coefficient (>0.3 considered adequate) was calculated between BIS and appearance scale of the EORTC QLQ-C30 to test convergent validity and pain, financial difficulties, pain killer, swallowing and/coughing scales of the EORTC QLQ-C30 and EORTC QLQ-HN35 measures to test discriminant validity (Gorecki, 2011). Criteria were used as guides to the magnitude of correlations, as opposed to pass/fail benchmarks (high correlation *r* > 0.7; moderate correlation *r* = 0.3–0.7; low correlation <0.3).

An analysis of variance (ANOVA) was used to estimate mean differences between Tamil, Telugu, and Hindi speaking HNC patients, on body image.

## Results

### Participants

A total of 621 participants, of which 205 were Tamil speaking, 216 Telugu speaking, and 200 Hindi speaking, completed all PROMs. Table 1 presents participants’ demographics for all three languages. The overall mean age of respondents was 50 years (range 19–89) and most were male (81.5%), married (86.3%), and employed (64.4%). About 84.9% reported substance use: either tobacco (chewing and/or smoking) and/or alcohol.

**TABLE 1:** Socio demographics for 621 patients across all languages.

Socio demographics	Tamil (*n* = 205)	Telugu (*n* = 216)	Hindi (*n* = 200)	Total (*n* = 621)
Age: Mean, Median (Range)	52.83, 54 (19–82)	50.5, 49 (21–89)	45.98, 45 (21–75)	49.77, 49 (19–89)
Missing	1	3	0	4
**Gender *n* (%)**				
Male	163 (79.5)	170 (78.7)	173 (86.5)	506 (81.5)
Female	41 (20)	42 (19.4)	26 (13)	108 (17.5)
Missing	1 (0.5)	4 (1.9)	1 (0.5)	6 (1)
**Marital Status *n* (%)**				
Married/De facto	183 (89.2)	169 (78.2)	184 (92)	536 (86.3)
Divorced/Separated	3 (1.5)	7 (3.2)	1 (0.5)	11 (1.7)
Single	6 (2.9)	8 (3.7)	13 (6.5)	27 (4.4)
Widowed	11 (5.4)	27 (12.5)	2 (1)	40 (6.4)
Missing	2 (1)	5 (2.3)	0	7 (1.1)
**Education qualification *n* (%)**				
Primary level	60 (29.3)	127 (58.8)	74 (37)	261 (42)
Intermediate level	78 (38)	28 (13)	46 (23)	152 (24.5)
Higher secondary level	39 (19)	27 (12.5)	26 (13)	92 (14.8)
Diploma	10 (4.9)	21 (9.7)	0 (0)	31 (5)
University degree	14 (6.8)	10 (4.6)	44 (22)	68 (11)
Post-graduate degree	1 (0.5)	3 (1.4)	9 (4.5)	13 (2.1)
Missing	3 (1.5)	0	1 (0.5)	4 (0.6)
**Employment *n* (%)**				
Employed	141 (68.7)	106 (49.07)	153 (76.5)	400 (64.4)
Unemployed	23 (11.2)	80 (37)	20 (10)	123 (19.8)
Homemaker	23 (11.2)	20 (9.2)	17 (8.5)	60 (9.7)
Training/education	1 (0.5)	2 (0.9)	4 (2)	7 (1.1)
Retired	1 (0.5)	1 (0.5)	5 (2.5)	7 (1.1)
Unable to work	13 (6.3)	1 (0.5)	0 (0)	14 (2.2)
Other	0	1 (0.5)	1 (0.5)	2 (0.3)
Missing	3 (1.5)	5 (2.3)	0	8 (1.3)
**Type of Cancer *n* (%)**				
Oral Cavity	94 (45.8)	108 (50)	181 (90.5)	383 (61.7)
Salivary Gland	2 (0.9)	6 (2.8)	1 (0.5)	9 (1.4)
Oropharynx	26 (12.6)	3 (1.4)	2 (1)	31 (5)
Larynx + Hypopharynx	35 (17.1)	9 (4.2)	0 (0)	44 (7.1)
Sinus gland	2 (0.9)	1 (0.5)	0 (0)	3 (0.5)
Tongue	35 (17.1)	35 (16.2)	9 (4.5)	79 (12.7)
Nasopharynx	9 (4.4)	7 (3.2)	0 (0)	16 (2.6)
Nasal Cavity	0	29 (13.4)	4 (2)	33 (5.3)
Unknown primary	1 (0.5)	11 (5.1)	0 (0)	12 (1.9)
Missing	1 (0.5)	7 (3.2)	3 (1.5)	11 (1.8)
**Stage of disease *n* (%)**				
I	3 (1.4)	8 (3.7)	14 (7)	25 (4)
II	34 (16.5)	24 (11.1)	63 (31.5)	121 (19.5)
III	114 (55.6)	69 (31.9)	84 (42)	267 (42.9)
IV	43 (20.9)	19 (8.8)	31 (15.5)	93 (14.9)
Missing	11 (5.3)	96 (44.4)	8 (4)	115 (18.5)
**Treatment offered *n* (%)**				
Radiation	205 (100)	177 (81.9)	199 (99.5)	581 (93.6)
Chemotherapy	198 (96.6)	121 (56)	33 (16.5)	352 (56.7)
Surgery	29 (14.1)	32 (14.8)	115 (57.5)	176 (28.3)
**Treatment status *n* (%)**				
Undergoing treatment	104 (50.7)	177 (81.9)	189 (94.5)	470 (75.7)
Survivor	97 (47.3)	39 (18.1)	11 (5.5)	150 (24.1)
**Substance usage *n* (%)**				
Yes	150 (73.2)	187 (86.5)	190 (95)	527 (84.8)
No	54 (26.3)	20 (9.2)	9 (4.5)	83 (13.3)
Missing	1 (0.5)	9 (4.1)	1 (0.5)	11 (1.7)
**ECOG Performance Status *n* (%)**				
0	51 (24.8)	25 (11.6)	99 (49.5)	175 (28.1)
1	110 (53.6)	75 (34.7)	77 (38.5)	262 (42.2)
2	23 (11.2)	71 (32.9)	17 (8.5)	111 (17.9)
3	16 (7.8)	26 (12)	5 (2.5)	47 (7.6)
4	4 (1.9)	13 (6.0)	1 (0.5)	18 (2.9)
Missing	1 (0.4)	6 (2.8)	1 (0.5)	8 (1.3)

### Data Quality

The criteria were satisfied for most psychometric properties evaluated. Data quality was generally high (scale scores were computable for 98–100% of respondents). Scale-to-sample targeting was good (scale scores spanned the scale range, were not skewed and values were within ±1.0); mean scores were near the scale mid-point for Hindi and Telugu but not the Tamil version, and floor effects exceeded the 15% criterion for Tamil version (see Table 2). The lowest and highest scores for BIS across all three languages were 0 and 30, respectively.

**TABLE 2:** Scale level analyses–data completeness and targeting of BIS across Tamil, Telugu, and Hindi (*n* = 621).

Scale	Data completeness–Computable scale score (%)	Targeting						
		Possible score range*	Observed score range	Range mid-point	Mean score	SD	F/C effect (%)	Skewness
**Tamil**								
BIS	99.0	0–30	0–30	15	9.28	10.066	32.0/5.4	0.808
**Telugu**								
BIS	100	0–30	0–30	15	12.756	7.685	2.9/6.7	0.583
**Hindi**								
BIS	98.0	0–30	0–30	15	12.41	7.363	1.5/4.6	0.757

*High scores indicate great bother/impact; SD standard deviation; F/C floor/ceiling–floor effect = % scoring 100 (greatest bother/impact); ceiling effect = % scoring 0 (least bother/impact); BIS, Body image scale.

### Factor Structure

The suitability of data for EFA were assessed and confirmed: Tamil (KMO = 0.950; Bartlett’s χ2 = 2561.939, *P* = 0.000); Telugu (KMO = 0.837; Bartlett’s χ2 = 1275.547, *P* = 0.000); Hindi (KMO = 0.863; Bartlett’s χ2 = 1747.382, *P* = 0.000). Correlation matrices revealed all the correlation coefficients were above 0.3, suggesting the EFA results could be considered.

The EFA results (Supplementary Table 1) revealed the presence of only one component with eigenvalue exceeding 1, explaining cumulative variances of 78.66, 65.91, and 71.42% for Tamil, Telugu, and Hindi versions, respectively. Hence, this one-factor was retained across all three languages as it was practically relevant and conceptually equivalent to the factor analysis of the original measure (Hopwood et al., 2001).

### Scale Reliability

The reliability coefficients (Cronbach’s alpha) for the BIS across all three languages were within the satisfactory range (>0.70) with values of 0.969 (Tamil), 0.919 (Telugu), and 0.891 (Hindi) (Table 3).

**TABLE 3:** Body image scale level analyses–reliability and scaling assumptions: validity within-scale analysis of measures across Tamil, Telugu, and Hindi (*n* = 621).

Scale	Cronbach’s alpha	Mean IIC	IIC range	Scaling assumptions–Corrected ITC range
				
**Tamil** BIS (*n* = 203)	0.969	0.928	0.530–0.913	0.722–0.941
**Telugu** BIS (*n* = 216)	0.891	0.449	0.220–0.840	0.433–0.743
**Hindi** BIS (*n* = 196)	0.916	0.528	0.348–0.983	0.611–0.753

*CI, confidence interval; IIC, inter-item correlation; ITC, item-total correlation (corrected for overlap); BIS, Body image scale.*

### Validity

Scaling assumptions were satisfied. Corrected ITC ranged from 0.722 to 0.941 for Tamil BIS, 0.433 to 0.743 for Telugu BIS, and 0.611 to 0.753 for Hindi BIS; see Table 3. Convergent and discriminant validity of the translated versions of the BIS were demonstrated.

### Convergent Validity

Correlations between BIS and the hypothesized item in EORTC QLQ HN-35 on appearance “*has your appearance bothered you?”* were consistent with the predicted moderate positive correlations (*r* > 0.30) across all three languages (see Table 4).

**TABLE 4:** Scale level analyses–convergent and divergent validity across Tamil, Telugu, and Hindi.

Scale	Convergent validity	Discriminant validity					
	QLQ-HN35 Appearance item *r*^1^	QLQ-HN35 Pain scale *r*^1^	QLQ-HN35 Swallowing scale *r*^1^	QLQ-HN35 Pain killers scale *r*^1^	QLQ-HN35 Coughing scale *r*^1^	QLQ-C30 Pain scale *r*^1^	QLQ-C30 Financial difficulties scale *r*^1^
**Tamil** BIS (*n* = 203)	0.425*	0.380*	0.129	0.140*	−0.006	0.275	0.151*
**Telugu** BIS (*n* = 216)	0.466*	0.061	0.158*	0.045	0.104	0.268*	0.245*
**Hindi** BIS (*n* = 196)	0.437*	0.121	0.140	0.083	0.214	0.183*	0.041

*p = 0.05; BIS, Body image scale.

### Discriminant Validity

Correlations between BIS and the scales of EORTC QLQ-HN35 (pain, swallowing, pain killers, and coughing) and EORTC QLQ-C30 (pain and financial difficulties) across all three languages were consistent with predictions (*r* < 0.30); see Table 4, thus supporting those responses to BIS are not biased by pain, coughing, swallowing, or financial difficulties.

### Body Image Between Different Language Groups

The effect of different language groups and their respective cultures on body image were statistically tested with one-way ANOVA. We found a statistically significant difference (*F* = 11.0954, *P* < 0.05) between means of Tamil, Telugu, and Hindi groups, with higher body image scores in Telugu (*M* = 12.86; *SD* = 7.65) and Hindi groups (*M* = 12.52; *SD* = 7.36) indicating more symptoms/body image distress, when compared to Tamil speaking population (*M* = 9.28; *SD* = 10.04) (see Table 5).

**TABLE 5:** Body image between Tamil, Telugu, and Hindi language groups.

Language groups	*n*	Mean	SD	*F*	*p*-value
Tamil	203	9.28	10.04	11.0954	0.00001854*
Telugu	216	12.86	7.65		
Hindi	196	12.52	7.36		

*p-value < 0.05; SD, Standard deviation; F = ANOVA.

## Discussion

The incidence of HNCs is increasing in global regions such as India. Treatment for HNCs commonly leads to significant changes to appearance and negative effects to one’s body image. The BIS is a reliable and valid PROM for measuring body image in cancer patients. Studies have reported BIS has content appropriate to patients with breast cancer, HNC, colorectal cancer, and benign gynecological conditions (Brédart et al., 2007). However, it is a screening measure, that does not indicates a specific diagnosis related to body image disturbance. We recently translated the BIS into three common Indian languages (Shunmugasundaram et al., 2021). This study assessed the psychometric properties of the translated Indian versions of the BIS in a large sample of HNC patients.

The Tamil, Telugu, and Hindi versions of the BIS satisfy criteria for reliability and validity in line with recommended Food and Drug Administration guidelines for PRO instruments (Speight and Barendse, 2010). Thus, these translated versions can be used to assess body image in Indian HNC populations’ in future clinical research and in clinical practice.

In general, our findings are comparable with the original BIS validation results and other studies of language translations (Hopwood et al., 2001; Moreira et al., 2010; Karayurt et al., 2015). For construct validity of the Indian versions of the BIS, we found support for the factor structure (Hopwood et al., 2001; Karayurt et al., 2015). Kaiser’s criterion was used to determine the number of factors extracted. A one-factor solution for the BIS has emerged in both EFA and CFA, explaining 57.55, 61.8, and 68.11% of the total variance, respectively, consistent with other studies (Hopwood et al., 2001; Karayurt et al., 2015).

Our scaling findings were within acceptable criteria and convergent and discriminant validity was demonstrated from confirmed hypotheses about constructs expected to be related or unrelated. These findings are consistent with the original validation study, and also with the Portuguese and Turkish translation studies (Hopwood et al., 2001; Moreira et al., 2010; Karayurt et al., 2015). A BIS Portuguese validation study evaluated convergent validity and found moderate to high correlations between the Experience of Shame Scale’s body shame scale and Derriford Appearance Scale–24 consciousness of appearance scale with BIS (Moreira et al., 2010). In the same study, discriminant validity was supported by low correlations between BIS and WHOQOL (Physical, Environmental, General facet) scales (Moreira et al., 2010).

Reliability is the overall consistency of a PROM which can be calculated either by internal consistency of the items or test–retest reliability. High levels of internal consistency reliability provide greater confidence when using a measure to compare different groups (for example, those undergoing different treatments or those with different diagnoses). The reliability coefficients for all three Indian language versions were supported, with all Cronbach Alpha’s >0.89. These findings are consistent with those reported for the original BIS (0.93), Portuguese version (0.92–0.93), and Turkish version (0.94) (Moreira et al., 2010; Karayurt et al., 2015). These findings suggest the Indian versions of the BIS are equivalent with the original and other language translations in terms of content (Shunmugasundaram et al., 2021), reliability, and validity.

Data completeness was high for all three Indian language groups indicating there were no items to which a high proportion of participants did not respond. This is important as high levels of non-response can indicate problems with an item such as difficult to understand, or the measure contains distressing or irrelevant content. Generally, a total of <30% respondents selecting “not at all” or “very much” indicates an item does not show significant “floor” or “ceiling” effects, respectively. Analysis of response distributions showed all response categories were used for all items with no significant floor or ceiling effects in Telugu and Hindi populations. However, floor effects (32%) were found in the Tamil population. These results may be a reflection of the sample as only 50% of the Tamil samples were undergoing treatment, unlike Telugu (82%) or Hindi (94.5%) sample, and subsequently experiencing less impact on body image. Cognitive debriefing interviews conducted during the translation and linguistic validation phase with a diverse HNC patient population found the BIS was relevant, comprehensive, and unambiguous (Shunmugasundaram et al., 2021).

Our findings suggest Tamil speaking HNC population have fewer body image concerns than Telugu and Hindi speaking groups. Previous literature reports body image concerns were significantly higher among HNC patients with speech and eating concerns (Rhoten et al., 2013). Since, 81.9 and 94.5% of our Telugu and Hindi speaking groups were undergoing treatment, they may have been experiencing these concerns during their participation in the study. Studies have also found non-surgically treated patients with HNC have a better body image outcome than surgically treated patients (Rhoten et al., 2013; Ellis et al., 2019; Sundaram et al., 2019). Since 57.5% of the Hindi speaking population reported undergoing surgery for their HNC treatment, their body image dissatisfaction could be associated to visible disfigurement from surgery. Few studies have examined body image in Indian populations treated for HNC. Qualitative studies in the future should explore experiences of patients with HNC in India, with a focus on body image.

Body image is an important area of HRQoL research and about 75% of HNC patients treated surgically experience some level of bodily changes, often resulting in psychosocial challenges (Nayak et al., 2016). Although most common reactions to bodily changes are poorer body image and self-esteem and increased distress, self-consciousness and anxiety, some patients experience positive impacts such as regained confidence from their scars and view their scars as a reminder to be grateful for being alive and surviving cancer (Manier et al., 2018). India is a culturally diverse country and the floor effects in this study reflect that. Hence, it is possible that sample demographics and culture contribute to Tamil speaking participants low score on body image (low scores indicate no appearance related concerns).

Current globalization trends and the increasing importance of multinational clinical trials highlight the need for PROMs in languages other than English. PROMs developed in western countries may not be applicable to other cultures and require cross-cultural and linguistic validation. Although adapting a PROM for a different cultural group can be arduous and requires considerable investment of time and resources, unless this process is undertaken, the results of research using that PROM may be questionable. This is because language and culture have an impact on the way people respond to questions (Halliday and Hasan, 1989; Shunmugasundaram et al., 2021). If a non-English speaker answered a PROM in English, their responses may be biased or inaccurate, limiting the quality of the data. We need multilingual PROMs, suitable for routine clinical and research use, which are brief and inflict minimal patient burden to enable assessment of important outcomes and possibly improving study participation rates among ethnic groups in English speaking countries (Gupta and Padhy, 2011).

There is strong evidence that psychosocial interventions improve outcomes in cancer patients. However, for interventions to be effective, they need to be tailored to disease-specific outcomes. Using disease-specific measures will capture outcomes appropriately, particularly if they are translated and validated across languages and cultures. Assessing important outcomes could be used in clinical practice to help healthcare professionals identify concerns and address them directly. Although substantial efforts with regards to PROM development and validation have been made in the western literature to ensure robust measures are available, less has been studied and found available in the Indian literature (Saxena et al., 1998; Shah et al., 2016). This work serves as a model for ensuring future cultural relevance of measures for use in multinational trials.

This study had a number of strengths such as large samples across the three language groups, representative of different HNC types, disease stages and treatment phases, from multiple centers across India, and psychometric analyses to test the reliability and validity of the BIS according to internationally accepted standards. Future work will evaluate clinical validity (known groups) and responsiveness of the Indian versions of the BIS in longitudinal studies and the psychometric properties of the Tamil, Telugu, and Hindi versions of BIS among Indian immigrant populations with HNC living abroad. As India is a diverse country with a range of cultures and languages, more language translations or cross-cultural adaptations may be required to ensure inclusion of the entire Indian population in multinational trials.

## Conclusion

The Tamil, Telugu, and Hindi versions of the BIS are valid and reliable measures of body image, with equivalent content and psychometrics as the original version as well as other language translations. The Indian BIS versions can be used in both clinical research and in healthcare settings to assess body image disturbances across HNC disease and treatments in Indian HNC patients in India and abroad.

## Author’s Note

This study formed part of CS’s PhD work.

## Data Availability Statement

The raw data supporting the conclusions of this article will be made available by the authors, without undue reservation.

## Ethics Statement

Ethics approvals were obtained from the University of Sydney Human Research Ethics Committee (Sydney, Australia; Ref: 2019/202), Scientific Advisory Committee and Ethical Committee of Cancer Institute, WIA (Ref: IEC/2019/Sep 02), Ethical Committees of MNJ Institute of Oncology and Regional Cancer Centre (Regd No: ECR/Inst/AP/2013/RR-16 dated 18/04/2019; no reference number was provided in the letter of approval), and Nanavati Super Specialty Hospital. The patients/participants provided their written informed consent to participate in this study.

## Author Contributions

CS recruited participants and collected data (in one site) and supervised recruitment and data collection in the other sites, entered and managed data, checked for accuracy, analysed, and interpreted the findings, drafted, and wrote the manuscript. CR, HD, PB, and PS conceived the presented idea, developed the study design, helped plan the study and contributed to the preparation including ethics and data collection and supervised the overall study. MC, SV, NA, and NH contributed to ethics, recruitment, data collection, and discussed the results and contributed to the final manuscript. CR and HD verified the analytical methods and supervised the findings of this work. All authors contributed to the article and approved the submitted version.

## Conflict of Interest

The authors declare that the research was conducted in the absence of any commercial or financial relationships that could be construed as a potential conflict of interest.

## Publisher’s Note

All claims expressed in this article are solely those of the authors and do not necessarily represent those of their affiliated organizations, or those of the publisher, the editors and the reviewers. Any product that may be evaluated in this article, or claim that may be made by its manufacturer, is not guaranteed or endorsed by the publisher.
