# Supplementary material for: Body Image Scale: Evaluation of the Psychometric Properties in Three Indian Head and Neck Cancer Language Groups
Source: Front Psychol. 2022 May 12;13:779850. doi: 10.3389/fpsyg.2022.779850 (PMC9135105; doi:10.3389/fpsyg.2022.779850)
Supplement: Supplementary file 1 [file Table_1.docx]

Supplementary Table 1 Body image scale items only: Principal Component analysis (EFA) with Varimax rotation

| Item | **Factors and loadings (exploratory factor analysis)^a^** | | |
| --- | --- | --- | --- |
|  | Tamil  Factor | Telugu  Factor | Hindi  Factor |
| BIS_1 | 0.834 | 0.761 | 0.773 |
| BIS_2 | 0.892 | 0.806 | 0.812 |
| BIS_3 | 0.928 | 0.818 | 0.783 |
| BIS_4 | 0.927 | 0.758 | 0.723 |
| BIS_5 | 0.901 | 0.771 | 0.740 |
| BIS_6 | 0.944 | 0.684 | 0.713 |
| BIS_7 | 0.953 | 0.621 | 0.687 |
| BIS_8 | 0.766 | 0.511 | 0.777 |
| BIS_9 | 0.906 | 0.672 | 0.789 |
| BIS_10 | 0.797 | 0.687 | 0.783 |

**^a^** BIS – Body image scale
